# Deep transfer learning with multimodal embedding to tackle cold-start and sparsity issues in recommendation system

**DOI:** 10.1371/journal.pone.0273486

**Published:** 2022-08-25

**Authors:** Syed Irteza Hussain Jafri, Rozaida Ghazali, Irfan Javid, Zahid Mahmood, Abdullahi Abdi Abubakar Hassan

**Affiliations:** 1 Faculty of Computer Science and Information Technology, Universiti Tun Hussein Onn Malaysia, Parit Raja, Malaysia; 2 Department of Computer Science and Information Technology, University of Poonch, Rawalakot, AJK, Pakistan; 3 Department of Computer and Information Technology, University of Kotli Azad Jammu and Kashmir, Kotli, AJK, Pakistan; Hanyang University, REPUBLIC OF KOREA

## Abstract

Recommender systems (RSs) have become increasingly vital in the modern information era and connected economy. They play a key role in business operations by generating personalized suggestions and minimizing information overload. However, the performance of traditional RSs is limited by data sparseness and cold-start issues. Though deep learning-based recommender systems (DLRSs) are very popular, they underperform when considering rating matrices with sparse entries. Despite their performance improvements, DLRSs also suffer from data sparsity, cold start, serendipity, and generalizability issues. We propose a multistage model that uses multimodal data embedding and deep transfer learning for effective and personalized product recommendations, and is designed to overcome data sparsity and cold-start issues. The proposed model includes two phases. In the first—offline—phase, a deep learning technique is implemented to learn hidden features from a large image dataset (targeting new item cold start), and a multimodal data embedding is used to produce dense user feature and item feature vectors (targeting user cold start). This phase produces three different similarity matrices that are used as inputs for the second—online—phase to generate a list of top-n relevant items for a target user. We analyzed the accuracy and effectiveness of the proposed model against the existing baseline RSs using a Brazilian E-commerce dataset. The results show that our model scored 0.5882 for MAE and 0.4011 for RMSE which is lower than baseline RSs which indicates that the model achieved an improved accuracy and was able to minimize the typical cold start and data sparseness issues during the recommendation process.

## 1 Introduction

Due to the rapidly increasing volume of data over the internet, the users often face difficulty finding the information of their interest. Users are often astounded by huge volumetric data which makes the decision making process more difficult for them [1]. To tackle this information overload challenge, researcher’s community has widely explored the domain of recommender systems in recent times. Recommender systems reduce the user load by producing automated recommendations for the items of user’s interest [2]. RSs provide a personalized shopping experience to the users by making effective predictions about items of user’s preference and help in improving the sales and attraction of users to e-commerce websites [3]. These systems strive to save the users’ time by suggesting appropriate items based on their preferences and decreasing their efforts in scanning a typically huge and unfamiliar item space [4].

Several algorithms have been developed to give consumers more effective, efficient, and accurate personalized recommendations based on their preferences [3]. Traditional techniques including Collaborative Filtering (CF) RSs, Content-based (CB) RSs, and Hybrid RSs have been explored by the researchers in RSs domain [5]. Content-based RS incorporate content of items for which, a user has expressed an interest. Collaborative filtering models are based on items favoured by people sharing similar tastes. To give effective and tailored recommendations, hybrid recommendation systems utilize both CB and CF-based recommendation algorithms [6]. However, combining CB and CF techniques to target cold-start and sparseness issues increases the algorithm’s computing cost and complexity [7].

Despite major advancements, RSs still have a few fundamental limits and flaws that need to be addressed and have piqued academics’ interest. Sparsity and the cold-start issues are examples of such restrictions. For example, rating data for many applications is relatively sparse or rating is not available for users (user cold-start) or objects (item cold-start) which compromises the quality of the recommendation process [8, 9]. Cold-start and sparsity are two prominent and hot issues in RSs, and numerous solutions have been presented [10, 11]. However, they struggle to deal with it effectively, particularly in circumstances of sparse input, such as when a high number of users and items exist but just a few people have rated each item [12]. Generating a profile of a user or an item is relatively a much complicated task. A decent profiling approach should include both explicit and hidden or latent features when describing a user [13]. Traditional recommendation methods are often unable to use these hidden information, resulting in poor suggestion performance.

In addition, deep learning’s influence is very widespread, with recent demonstrations of its usefulness in information retrieval and recommender systems [14]. Deep neural networks (DNNs) have lately been used by numerous recommender system researchers to improve suggestion performance. However, because these models predict user, and item features on user-item interactions [15, 16], they also underperform in the case of sparse user-item interactions [17]. Deep Transfer Learning (DTL) is another technique where we use a pre-trained deep learning model on small scale datasets to transfer and utilize the knowledge learnt from training these pre-trained models on relevant larger datasets earlier [18]. This allows transferring knowledge from pre-trained deep models which extends the capability of these models to produce efficient predictions and improved accuracy [19]. DTL has shown promising impacts in other domains of machine learning like NLP and computer vision, but is under-explored in the field of recommender systems [20].

Furthermore, user Metadata (user profile information, session logs, social network embedding) and item visual features (color, contrast, shape) are a rich source of relevant information which may be very helpful in the production of a dense user, and item feature matrices. Deep learning techniques have shown promising improvements in computer vision and NLP, and have been exploited in the domain of RSs as well. However, due to computational cost and complexities, DL methods face adoptability and generalizability issues in RSs. To tackle both user and item cold-start challenges, we propose a novel Deep Transfer Learning with Multi-modal Embedding (DTLME) based hybrid recommender model for producing effective recommendations after generating rich user and item profiles.

The proposed DTLME model deals with the data scarcity and cold-start issues with the help of two models. Firstly, Deep transfer learning (DTL) is applied for generating an item-item similarity matrix for alleviating new item-cold start issue. Secondly, multi-modal embedding (ME), is used for learning the latent features from multiple information sources such as user embedding, session logs, social network embedding and item embedding in addition to the rating matrix, to produce dense feature vectors and eliminate the concern of sparseness in the training data. The dense user and item profiles are then employed in a user-based collaborative recommendation system to find each user’s nearest neighbors. Following are the primary contributions of this work:

- Deep transfer learning approach to learn image latent features and compute item-item similarity matrix on the basis of visual similarities between items, in order to deal with new item-cold start problem.
- Producing dense user and item matrices by integration of multimodal embedding to learn better user profiles from user’s basic information, context information, and social network embedding in addition to the sparse rating matrix. Application of singular value decomposition (SVD) overcomes the sparsity issue and produces dense user-user and user-item similarity matrices.
- A hybrid recommendation model to produce dense similarity clusters based on K-nearest neighbors, produces an effective and personalized list of top-n recommendations for a specific user.
- Lastly, a list containing N top items is generated by combining *i* top items from item-item similarity matrix and *j* top items from similarity clusters produced during the recommendation process, where *i* and *j* are adjustable parameters given as part of the input to our algorithm.

The rest of the article is laid out as follows. Related literature is discussed in Section 2. The methodology and the proposed model are described in Section 3. Section 4 covers experimental details and outcomes of the suggested DTLME model. At last, the conclusion is given in Section 5.

## 2 Related literature

In this section, we firstly discuss some of the relevant work in the domain of RSs. Then the cold start issue and some of the prominent cold-start techniques are reviewed.

Various RSs have been developed for various application domains and are available in the literature. All RSs use algorithms to provide recommendations based on the data they have about their customers. These methods are based on the techniques for screening. Two types of filtering systems are utilized, Content-Based (CB) Filtering [21] and Collaborative Filtering (CF) [22] techniques. CF is a type of filtering in which multiple people work together to solve a problem. Meanwhile, filtering by content the notion is that if a person enjoys a product, he or she will recommend it to others. Other similar products are very likely to appeal to him or her. Collaborative filtering employs two methods: Latent Factor Model (LFM) [22] and a neighborhood-based approach built on LFM, [23]. The LFM-based strategy aims to identify elements that assist in understanding users’ personalities and preferences while recommending a product. In most cases, collaborative filtering outperforms content-based filtering when it is compared for performance and usability [21, 22]. Hybrid filtering systems also exist which combine CB and CF strategies to maximize the benefits of the two [22, 23].

Moreover, when a new item gets added to a RS’s repository or a fresh user gets engaged with a system, RS faces difficulty in making inferences about user interests; known as a cold-start challenge [24–26]. In RSs domain, there exist two types of cold-start problems: (1) a new user cold-start, and (2) a new item cold-start problem. A new user is presented to the system in the new user cold-start scenario, and RS has difficulty making recommendations because it lacks information about the individual. The system would have no rating score for a new item in the cold-start scenario, which makes it difficult to predict an interested user for the item. User cold-start is a more problematic challenge than that of item-cold start, and it has been extensively researched [17, 24–26].

The fundamental problem with the cold-start is the lack of information needed to produce recommendations. The proposed solutions outline means for gathering this information that is currently unavailable. The data can be gathered either overtly by questioning the user or implicitly by relying on previously obtained data. Researchers have made certain attempts and proposals to resolve the issues of cold-start. Abu-Salih *et al*. [27] used a meta-learning technique to solve a problem related to item-cold start, Sanchez *et al*. attempted to deal with this issue using an active learning model [28], doc2vec was used in [29] to solve an item cold-start issue, and Vartak *et al*. [30] used user’s demographic information to solve user cold-start issues. These works, on the other hand, have a number of flaws: for example, proposed technique [31] is computationally intensive since it trained two independent neural models, where the first one was used to learn generic representation for items and the other one was used to learn a sole representation for each category. Furthermore, test-time estimates take longer due to incompatibility with current meta-learning methods based on gradients [30], where, for rapid adaption, only a few gradient modifications are required. Similarly, technique [28] suffers from the same limitations as content-based filtering due to the requirement of additional item attributes to solve cold-start issues. In case, when there are not enough feedback and item attributes are not available, this approach cannot be used. The proposed model [29] deals with the employment recommendation only and cannot be applied to other recommendation domains. To tackle the cold-start problem, [30] depends on the personal demographic information of individuals, which is not always available. Furthermore, because the prediction engine is founded on simple hand-crafted properties, it is not relevant to a large range of activities [31]. Although the RecGAN [11] RNN-GAN based technique has been found to perform well in cold-start recommendations, there is no theoretical justification for that though.

To lighten the consequences of data sparsity, many modifications for user-based CF have already been proposed recently [11, 32]. A singular vector decomposition [33] was implemented to concentrate particular user matrices for dimensionality reduction, and similarity measurements [12] were applied for grouping users and objects on a similarity basis. These solutions, on the other hand, have the disadvantage of necessitating the updating of the decomposition each time a new user is added or a rating is introduced in a matrix. Another recent innovation [34], is based on predicting errors for improving accuracy in user-based CF. The cost of calculating the errors of all ratings during training is a disadvantage of this technique. Alternative approaches have been proposed that employ recursive prediction algorithms to make use not only of neighbors, but also their neighbors [35]. The strategies have higher processing costs because all neighbors’ similarity values are obtained. Furthermore, these solutions must augment the information within user-item co-relation matrices for improvements and effectiveness of a user-based technique [36]. Additionally, two different measures for item-based similarities in [37, 38] were created to overcome the cold-start challenge on well-known datasets like MovieLens by adding item genre data.

In order to create reliable predictions, clustering methods are prevalent in RSs. Clustering is a process to group a set of items based on their properties and aggregate them based on how similar they are. Clustering-based approaches have proven to surpass other similarity measurements in terms of discovering users who are closer to a targeted user. These methods can be helpful in assisting with challenges involving sparsity and higher dimensionality [39]. To group people based on their social information, a hierarchical clustering algorithm [40] was used, followed by typical collaborative filtering to grade projections. The authors adjusted the k value of clustering algorithms [41] to recommend movies to the user and used social network analysis to verify the quality of the recommendations.

Integrating embedding from side information along with the rating matrix can be more effective for extracting latent similarity information for users and items. SimilarMF, proposed in [42] used embedding and social information along with a rating matrix to produce user-user and item-item similarity matrices which showed improved results. Neural Social Recommendation [43], a deep model based on matrix factorization, makes use of social information with user embedding to exploit user, item latent features for improving the prediction process. To avoid sparsity in the graph, the Collaborative Similarity Embedding (CSE) technique [44] leveraged direct relations from the input graph to discover similarity matrices. To cope with sparse data, a similar study [45] implemented a deep neural network and matrix factorization (ME-DCR) in combination. A hybridized social RS based on deep learning was created [46] to solve the scarcity problem of CF by embedding social data into their proposed architecture. To handle the sparsity challenge in CF, Aljunid *et al*. [47] proposed a deep collaborative recommender system (DCLRS).

Furthermore, DCF (hybrid deep collaborative filtering system) was proposed in [48] to handle both, sparsity and the cold-start challenges of CF. To ameliorate the sparseness, a deep network system, stacked denoising auto-encoder [49], uses underlying vectors and side information. To retrieve item feature maps and alleviate the inherent sparsity of rating data, PMF was incorporated into a social network with RNN [50]. However, because the grading and opinions both are time-sensitive, and old opinions are seen as sparse data, they do not take into consideration any time-sensitive components or auxiliary information [51]. Despite various improvements, these systems only evaluate limited supplementary information about people and things, which has an impact on the quality of feature extraction and user profile development, and hence on the quality of recommendation [52]. In this work, we propose a novel hybrid recommender model for producing effective recommendations after generating rich user and item profiles which uses deep transfer learning and multimodal embedding’s at its core in the feature engineering phase to produce dense similarity matrices. The proposed modal targets both user cold-start and item cold-start problems at the same time by allowing the modal to list both familiar and non-familiar or new items in the top-n list of recommendations.

## 3 System architecture

**Fig 1:**
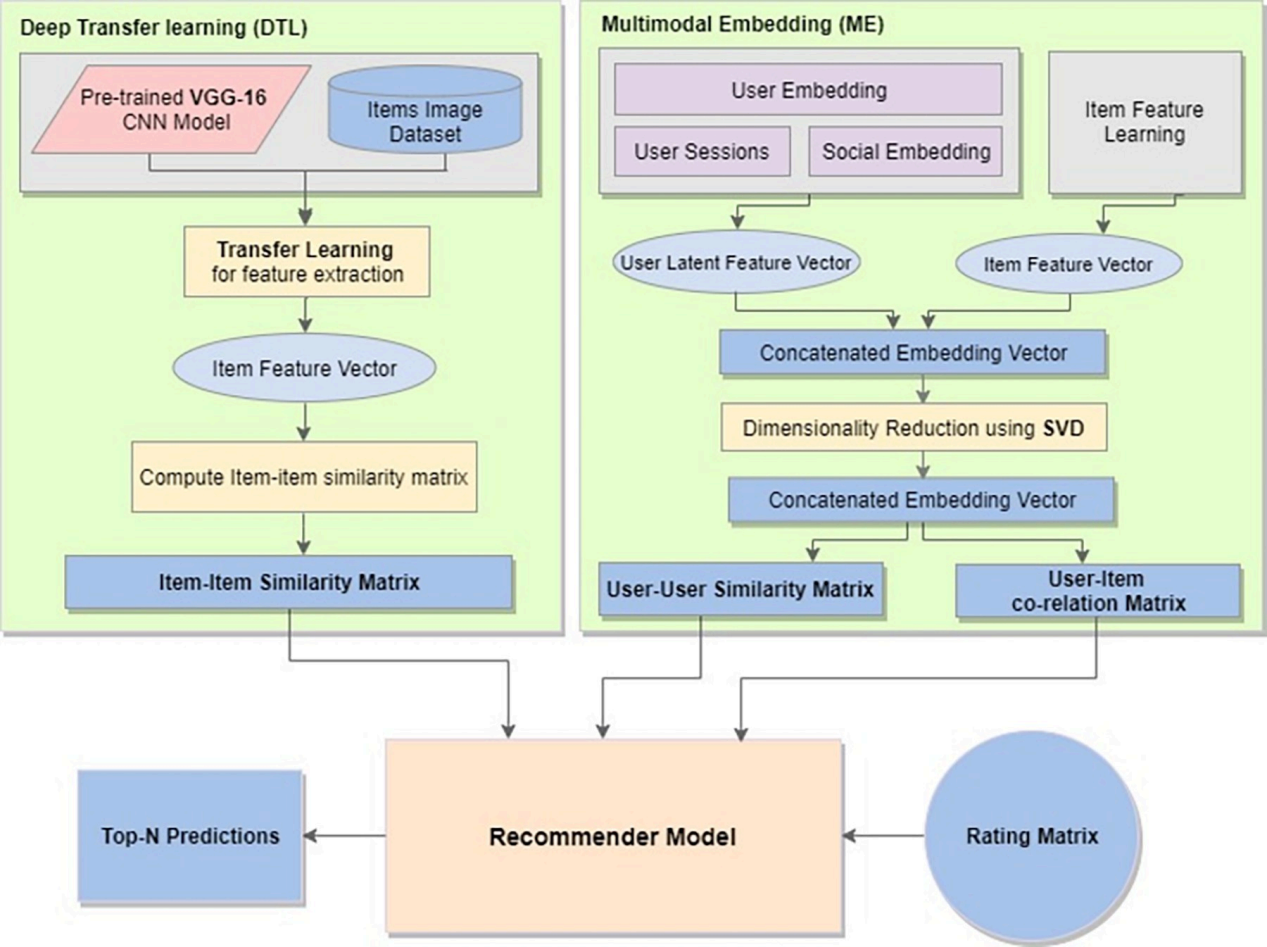
The architecture of the proposed DTLME model for feature engineering.

The proposed method basically works in two phases (Fig 1). The first phase is offline, which performs the feature learning on the dataset. In this phase, a transfer learning technique is applied to generate item latent feature vector from an image dataset [53] using VGG-16 [54], which is a convolutional neural network architecture also known as OxfordNet and named after the Visual Geometry Group at Oxford. The output of this model is an item based similarity matrix produced using cosine similarity computation. In the second part of this phase, multimodal data embedding is used to generate latent user-user similarity and user-item similarity matrix. The output of these two parts is then saved and further used as input to the recommender model in the next phase, which is performed online to produce effective recommendations for an active user.

### 3.1 Phase 1

The suggested model DTLME is described in detail in this section. We begin with an introduction of DTLME’s overall structure, followed by a detailed explanation of the model’s components.

#### 3.1.1 The architecture of the proposed model

Our proposed Deep Transfer Learning with Multimodal Embedding (DTLME) model comprises of two major components: 1) Deep transfer learning (DTL) for generating image features set from a large image dataset and the result is a similarity matrix by computing item-item similarity. 2) Multimodal Embedding (ME) is used for generating rich embedding vectors from multiple input sources and producing user and item co-relation matrices.

*3*.*1*.*1*.*1 Deep transfer learning (DTL)*. This module captures all of the images rich information as features. CNN is one of the most widely used and efficient deep models to extract maximum features from an image. However, training a CNN model on a selected dataset takes a huge time which is not favourable in the case of recommender systems. Transfer learning [14], is a deep learning approach that aims to improve machine learning performance by leveraging knowledge and other tasks completed by another machine learning system. The following are the arguments for utilizing pre-trained models: First, by employing the model for extracting information from an image corpus, learning can be transferred. Second, learning complex models on massive datasets necessitate increased computational resources. Third, learning the network takes a long time. As a result, we employ the DTL approach for extracting latent features that describe images.

**Fig 2:**
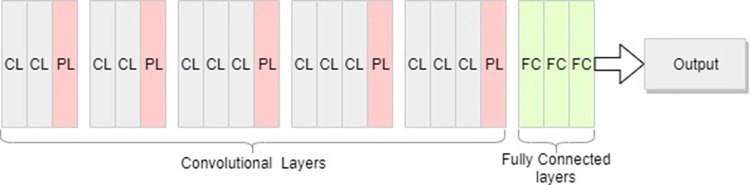
Generic VGG16 model.

We choose the VGG16 model, which has been shown to perform well on larger datasets and has a high level of precision. After deleting the corresponding output classification layer from the original pre-trained VGG16 model, we freeze the rest of the layers to minimize the loss of previous learning during the model execution. The generic architecture of the VGG16 model is given in Fig 2, where CL is the convolutional layer, PL is the pooling layer and FC is the fully connected layer.

**Fig 3:**
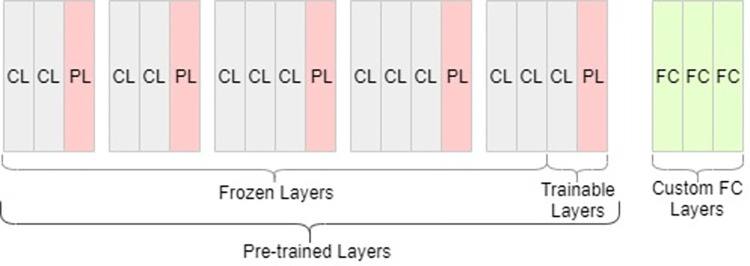
Pre-trained VGG16 model used for transfer learning.

The last two layers were left trainable including, fully connected and output layers and retrained the model to make predictions on our dataset using a process known as ‘fine tuning’. Custom fully connected layers are added to the end of this model for training and prediction. The pre-trained model produces a set of essential features which are saved as a flattened linear feature vector. We call this linear feature vector as “Items Feature Vector”. A similarity matrix is then computed using item-item similarity and the resultant matrix is saved which is later fed into our recommendation model for making predictions. The architecture of the pre-trained VGG16 model with frozen and trainable pre-trained layers is given in Fig 3.

CNN model is used as a classifier to predict the class of the input image, which increases the likelihood of receiving accurate recommendations. Using image classification for predicting target class saves the algorithm’s time, as similarity computations are performed against the predicted class items only. Cosine similarity measures are used for calculations of the item-item similarity using visual features to produce a similarity matrix.

*3*.*1*.*1*.*2 Multimodal Embedding (ME)*: ME module is further divided into sub-modules including, 1) User Feature Learning, 2) Item Feature Learning, 3) a feature reduction technique for reducing the dimensionality and complexity of the resultant embedding vectors to produce and linear embedding vector, and 4) generating user-user and user-item co-relation matrices from the resultant linear vector.

This component extracts the user and item relevant features from input data and produces an updated embedding feature vector as output after performing dimensionality reduction using a singular value decomposition (SVD) technique, to overcome the sparseness in the resultant feature vectors. Word2vec is used to generate an item feature vector from item metadata, along with the visual features of the item. User’s embedding vectors are produced from user basic information, session logs and social network embedding where social network embedding from Twitter and Facebook profile of the user is integrated into the feature learning process using Node2Vec model. Age, gender, ethnicity, profession, nationality, demographic location, and interests are the major attributes of the user which may participate in construction of rich user profile. The use of multimodal data embedding helps in building dense user profiles which could be very effective in alleviating user cold-start challenges.

**Fig 4:**
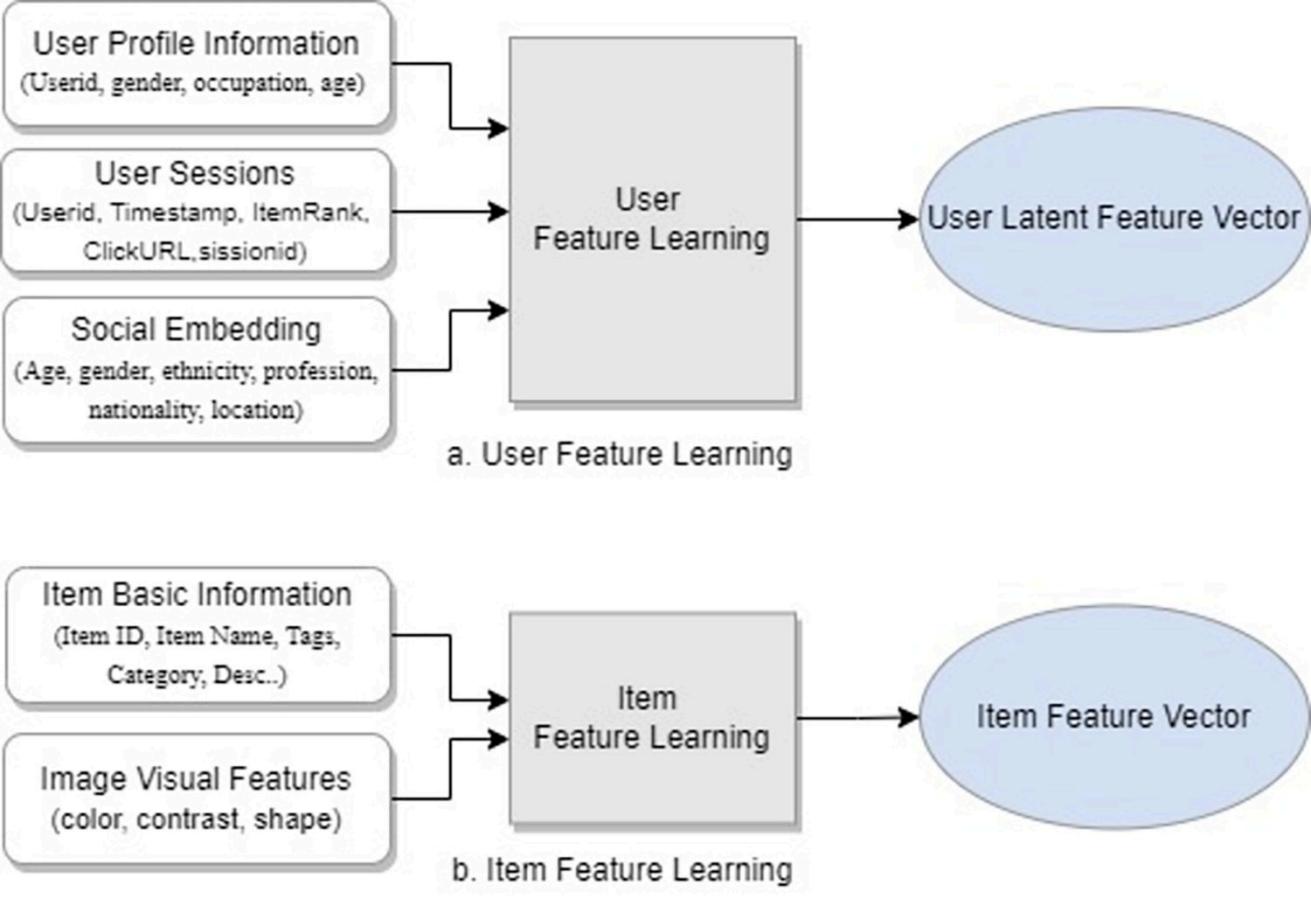
Multimodal embedding for feature learning. (a) User feature learning; (b) Item feature learning.

Fig 4 depicts the specific feature learning model, with part ***a***, representing user feature learning and part ***b***, representing the feature learning process for items. User profile information, user session logs and social profile of the user are used to generate dense user features set.

Combining side information with basic user information would be helpful in tackling the new user cold-start problem. Item latent feature vector is produced from the item’s basic information which will be later combined with the item’s visual features, extracted from the item’s image using the selected CNN model as discussed earlier.

*3*.*1*.*1*.*3 Similarity matrices*. Similarity matrices are produced after generation of latent feature vectors. When vectors are pointing in the same direction, cosine similarity is 1, 0 when they are perpendicular, and -1 when they are pointing in opposite directions. The range of values is -1 to 1, with -1 representing a most dissimilar and 1 representing a most similar.

If U denotes a group of users, u_i_ is the target user and u_j_ is another random user, P denotes a set of items, and p refers to an item from P, then user-user based, item-item based and user-item based similarity calculations are described below. The user-user similarity is calculated using ratings, to find out the similarity between the other users and the target user as shown in Eq (1).


$$Sim(u_{i},u_{j})=\frac{u_{i}.u_{j}}{\left|\left|u_{i}\right||*|\left|u_{j}\right|\right|}$$ (1)

Item-Item Similarity is calculated using the equation given below:

$$Sim(p_{i},p_{j})=\frac{p_{i}.p_{j}}{\left|\left|p_{i}\right||*|\left|p_{j}\right|\right|}$$ (2)

**Fig 5:**
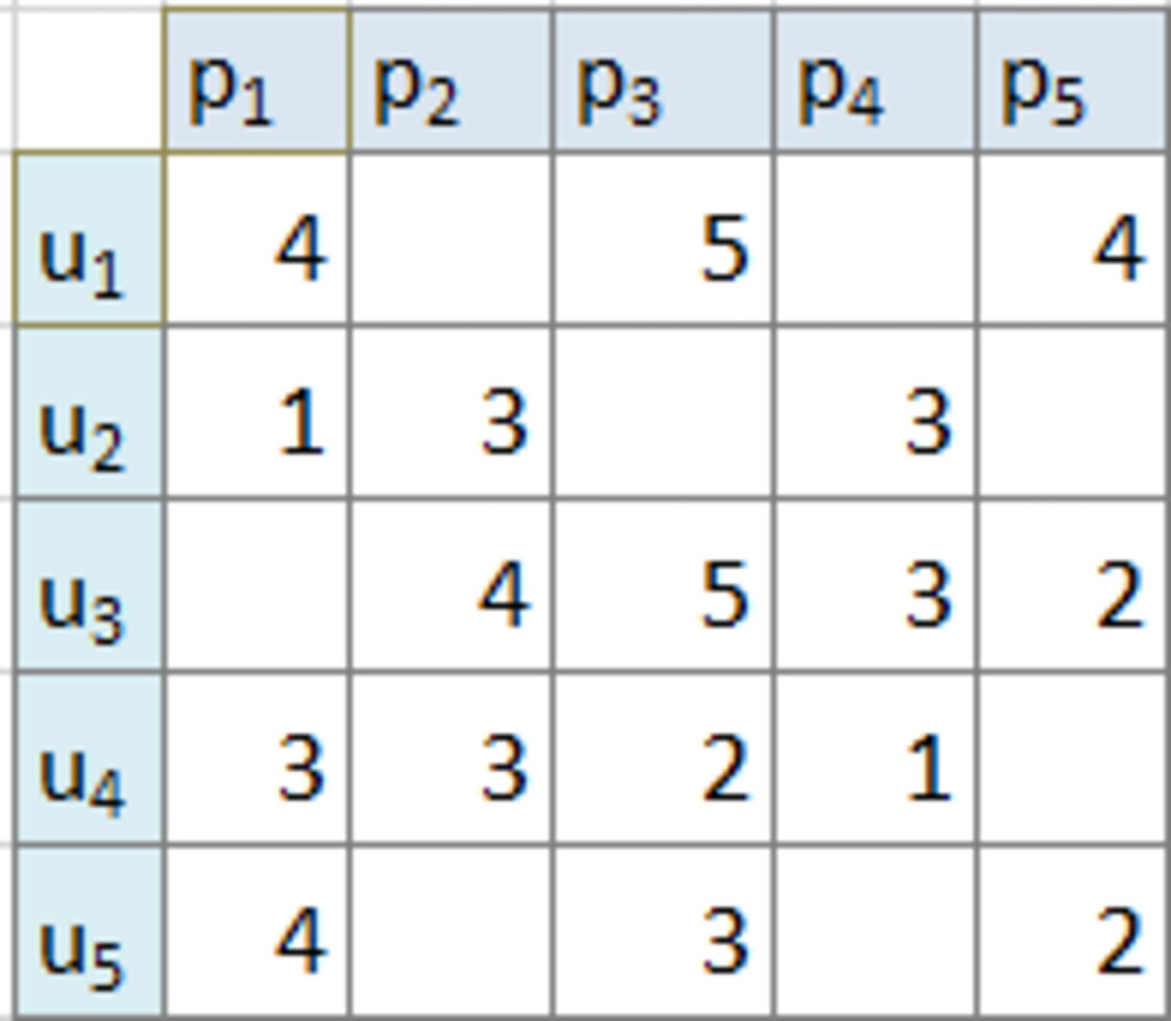
Sparse rating matrix for item *p*_*i*_ for user *u*_*i*_.

The rating matrix is often very sparse as newer products may have very few ratings or no ratings at all (Fig 5). Similarly, users are reluctant and they do not rate products usually. Another reason might be new users, having not ranked or rated any items and having no purchasing history as well. All these contribute to the sparseness of the rating matrix. Sparsity makes it difficult to identify the co-relations within users and between users and items. If X is a count of users and Y denotes a count of items, then the sparsity measure of the rating matrix can be calculated using Eq (3) below.


$$Sparsity=1-\frac{Total\;Ratings}{X*Y}$$ (3)

In order to overcome the sparsity issues in the rating matrix, we calculate the rating R that user U would be giving to an item p. Rating R for a user against P item is most likely to be close to the mean of P’s ratings by the top 5 or top 10 most similar users to that of U. The following mathematical model depicts an average rating for items, given by n users:

$$Ru=\frac{\sum_{u=1}^{k}Ru}{k}$$ (4)

**Fig 6:**
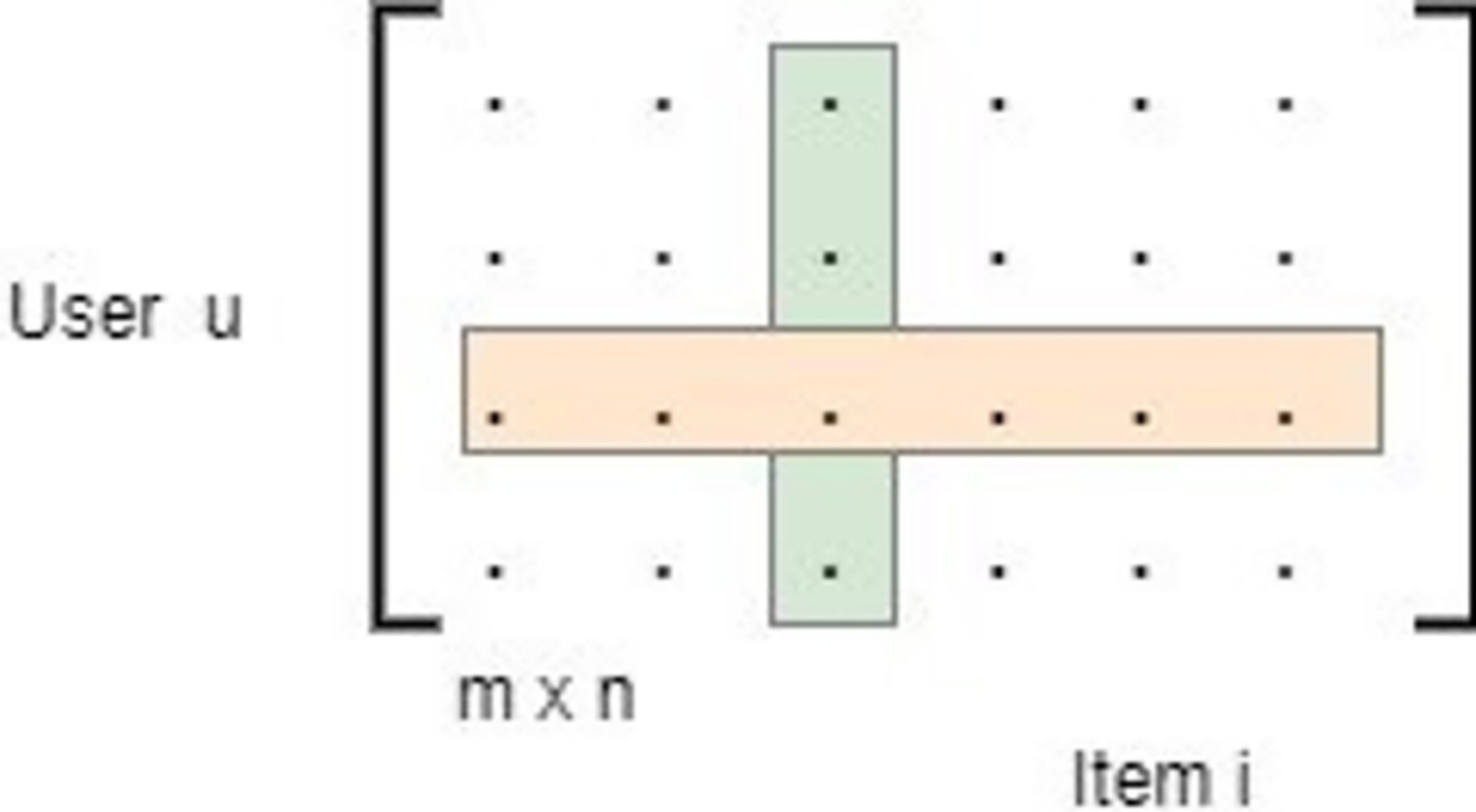
User-item affinity matrix.

A user-item co-relation matrix defines the affinity of a user to an item as shown in Fig 6. A dot product of user and item vectors is used to estimate a rating by the user to an item in affinity matrix.

User-Item Similarity is calculated using Eq (5):

$$Sim(U,K)=\frac{U.K}{\left|\left|U\right||*|\left|K\right|\right|}$$ (5)

### 3.2 Phase 2

**Fig 7:**
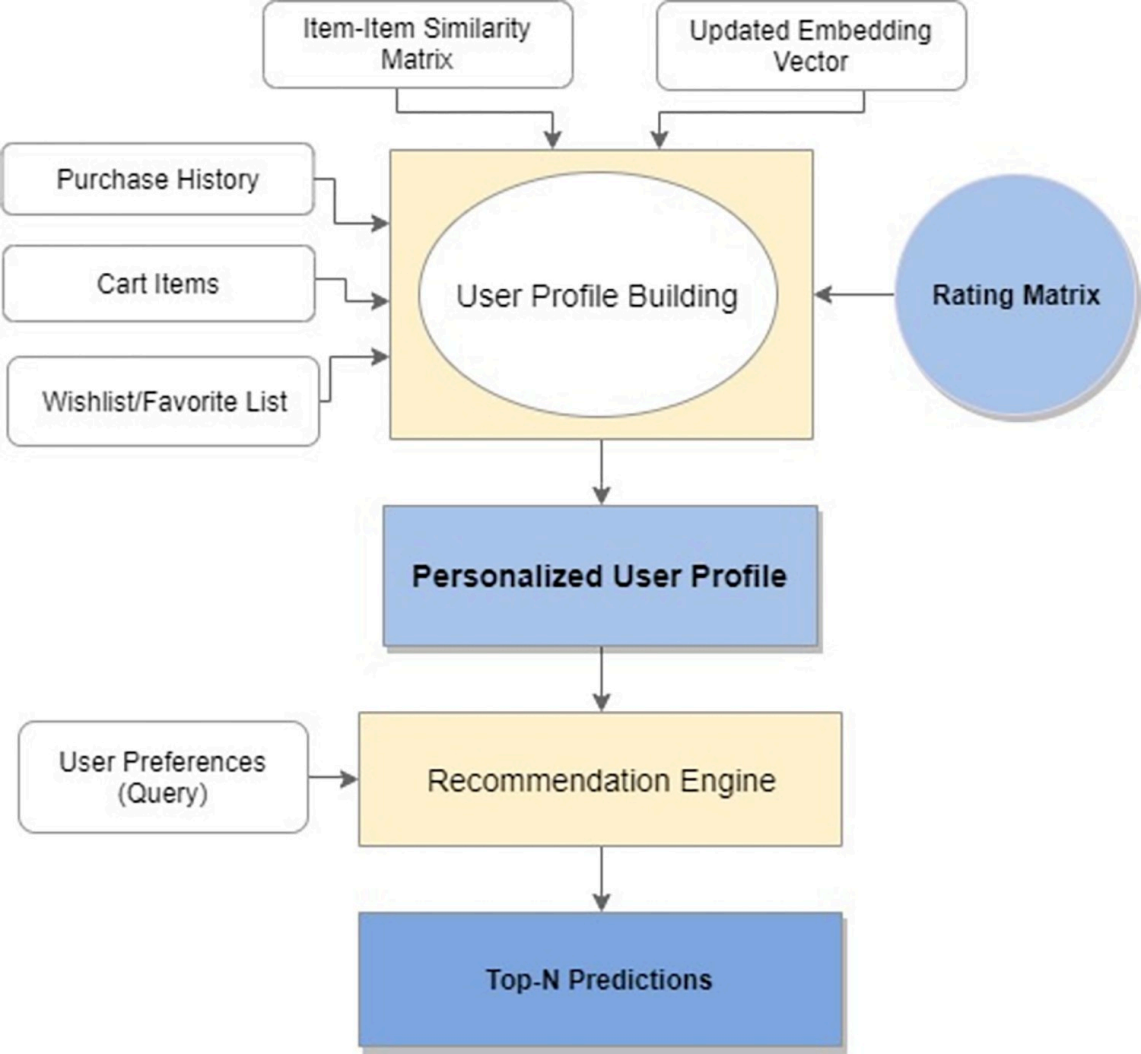
User profile, similarity calculation and top-n recommendation.

This phase entails making recommendations for an active user. It contains two main processes, 1) user profile building and 2) a recommendation module, to produce a top-N list of preferred items (Fig 7).

#### 3.2.1 User profile building and top-n recommendation

An effective and personalized user’s profile plays a vital role in improving the recommendation process. Our proposed model takes care of the necessary information about the user and item to generate a better user profile. Updated embedding vector and item-item similarity matrix from the previous step are fed to the user profile builder along with the rating matrix, user’s purchase history, shopping cart information and wish list items. User’s context information such as order history, cart items and wish-listed items help in the generation of more personalized user profiles for non-new users.

User profile information, along with the user preferences, is then passed onto the recommender model as input, which generates a list of top-n recommended items for an active user.

We perform the following two steps to cluster users on the basis of similarity measures and then try to predict the average rating for the new user.

1. Fetch ***N*** similar users to that of user ***u***, having a rating for an item ***i***.
2. Then, we estimate user ***u***’s rating for an item ***i*** by taking an average rating of ***N*** other users.

The process of calculating a rating for ***u*** from ***N*** identical users is represented in Eq (6).


$$R=\sum_{u=1}^{N}(SimilarityScore*rating)$$ (6)

### 3.3 Pseudo-code for the proposed model

**Input:** Target user ***u***_***t***_, Item feature vector, rating matrix ***R***, user embedding, item embedding, user session

information, social embedding, purchase history, cart items, wish-list items, ***n***, ***i***, ***j***

**Output:** Top-N recommended items for target user ***u***_***t***_

Compute dense rating matrix, initialize similarity matrices A, B, C, RecommendedItemsList, top-n

**a.** Transfer learning for visual feature learning from the image dataset

Load item feature vectors from image dataset using VGG-16 Pre-trained model

Perform fine-tuning if required (optional step)

Compute item-item similarity matrix A, for item feature vector using cosine similarity (2)

**b.** Multimodal embedding for user feature learning

Apply the word2vec model to learn word embedding from user context embedding, items context embedding and user session logs

Embed user’s social profile information using the node2vec model

Concatenate resultant embedding using a merge function to produce an updated embedding vector

Produce dense embedding matrix using SVD to eliminate sparsity from resultant vector

Compute similarity on the embedding vector to produce user-user similarity matrix B and user-item similarity matrix C using Eqs 1 and 3 discussed earlier.

**c.** Recommending top-n items to the user.

Inputs: A, B, C Matrices, user’s purchase history, shopping cart information, wish-list items, rating matrix, user preferences, ***n***, ***u***_***t*,**_ ***i*, *j***

For target user ***u***_***t***,_ build personalized user profile

Initialize RecItemsList

**While** len(RecItemsList)! = top-n

Find the subset of users ***u***_***s***_ who are most similar to an intended ***u***_***t***_

For user ***u***_***t***_, find a subset of relevant items using prediction score to identify user’s interest in an item based on user-user similarity

Add ***j*** number of items from a list of recommended items to RecItemsList

For the items vector, find a group of similar items to that of input item

Add ***i*** number of items from the recommendations to RecItemsList

**Output**: Return **Top-n** recommended items stored in RecItemsList.

## 4. Experimental design

A detailed analysis of the proposed model using two real-world datasets is covered in this section.

### 4.1 Datasets

Following datasets were used for feature learning, training and validation testing of the proposed model.

#### 4.1.1 Brazilian E-Commerce dataset

We used the Brazilian E-Commerce (BE-dataset) Public Dataset by Olist [53] for multimodal feature learning, a rich dataset containing the customer’s info, products information, order history, geo-location data, categories and order reviews. User sessions and social embedding information vectors are coupled with the latent features to generate rich user, item features vectors for making recommendations to the user. Table 1 presents the statistics of the BE-dataset for training and test sets used in our experiment.

**Table 1:** Training and testing set for Brazilian E-Commerce dataset.

	Users	Items	Orders	Ratings
Total Instances	99441	32951	98666	98410
Training Set	79552	26360	7893280	78728
Test Set	19888	6590	19733	19682

#### 4.1.2 E-Commerce product images

We have used the E-Commerce Product Images (Multi-label Data) [55] dataset for training of pre-trained VGG-16 CNN model and to produce latent feature vectors of the model using transfer learning technique. The dataset is divided into training data set with 14,720 images and a test dataset with 3000 images for validation as shown in Table 2.

**Table 2:** Training and testing set for E-Commerce product images dataset.

	Images	Labels
Total Instances	17,720	14290
Training Set	14,720	11336
Test Set	3000	2954

### 4.2 Evaluation metrics

We used precision as in Eq (7) to find out the number of recommendations for a particular user.


$$Precision=\frac{Ru}{TR}$$ (7)

Where *Ru* is the number of target user recommendations made by the proposed model. *TR* is a cumulative number of items that the model recommends.

Recall is the measure of correct recommendations made by the system [56]. The recall value is calculated using Eq 8 where *CR* denotes the correct recommendations and *TR* is total number of recommendations made by the model.


$$Recall=\frac{CR}{TR}$$ (8)

The formula for calculation of F-measure is given in Eq (9):

$$F-measure=\frac{2*P*R}{P+R}$$ (9)

Here, *P* stands for precision, and *R* denotes recall.

The average of the absolute deviations between predictions and actual data is called the Mean Absolute Error (MAE). It illustrates how far the projections were off the mark. The metric provides an estimate of the amount of the inaccuracy without indicating whether it is over, or under-predicted. A number of 0 means that there is no inaccuracy or that the forecasts are perfect.


$$MAE=\frac{\sum_{i=1}^{n}\left|ei\right|}{n}$$ (10)

Where, |*ei*| = |*pi*−*ai*|, *pi* represents value predicted and *ai* is original value.

To assess the performance of the experimental results with respect to the sparsity issue, the root mean squared error (RMSE), which is frequently used in RSs, is used. RMSE is calculated using Eq (11) below.


$$RMSE=\sqrt{\frac{1}{N}}$$ (11)

Where, *pi* is the predicted value, and *ai* is the observed value.

To assess the proposed model’s performance, item’s similarity was split into the training and test data sets. The proposed model was first tested on the training set to produce top-N recommendations and then Top-N items were compared with the list of predicted items from the test dataset to validate the model’s accuracy. The precision, recall, and F-1 measure [57] were used to assess the recommendation’s quality.

## 5. Results and analysis

### 5.1 Experiments

Following experiments were performed to validate the prediction accuracy and performance of the proposed model in comparison with other similarity based recommender systems. The experiments were carried out in Python programming language using Anaconda software and Jupyter notebook.

We adopted the VGG-16 pre-trained model for learning the image latent features from the E-Commerce Product Images dataset and then used the transfer learning technique to save the learnt feature vector in the form of a 2D array. ‘Adam’ was used as an optimizer function with cross-entropy as a loss function.

**Fig 8:**
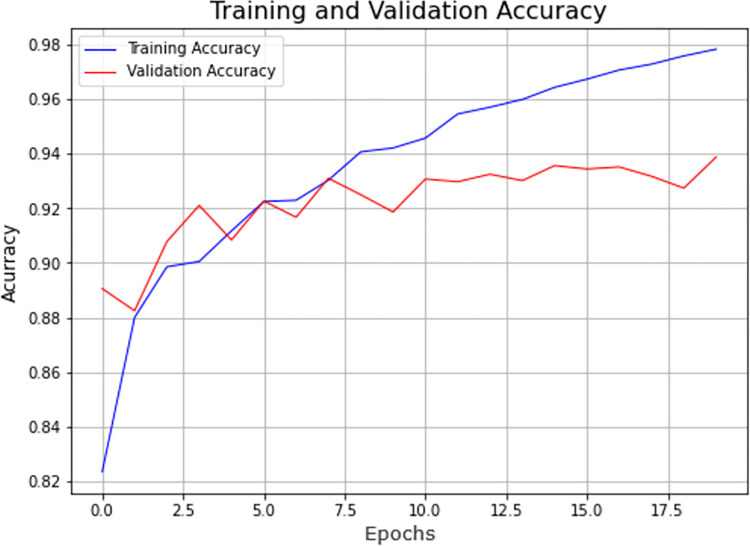
Accuracy measures for VGG-16 model.

**Fig 9:**
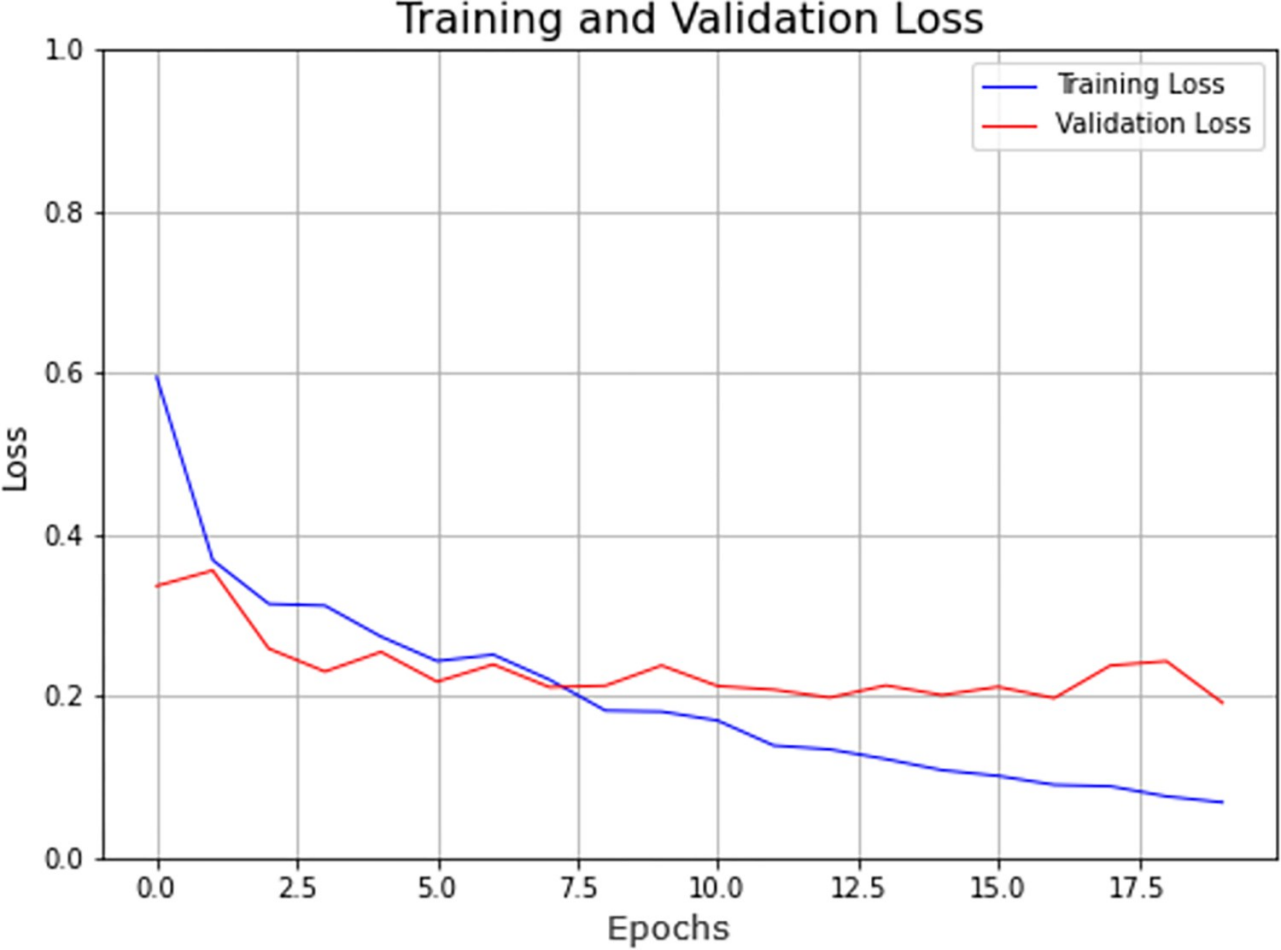
Loss for VGG-16 model.

The model achieved an accuracy of 0.9388 as shown in Fig 8, whereas, Fig 9 represents the value for its loss which was recorded at 0.1916 for 20 epochs with batch size 32 and a low learning rate.

After training the VGG-16 model on the selected dataset, the model was saved and loaded as a pre-trained model, freezing all the layers of the selected model and removing the last classification layer. To avoid the overfitting, the deep learning modal is fine-tuned on the selected dataset. A custom fully connected layer along with a dropout layer was added on the top of the model. Finally, the model was made non-trainable by setting its trainable parameter equal to false, i.e. *model*.*trainable = False*.

To address the issue of a new user’s cold start, a collaborative filtering technique was applied. For a particular user *u*, after creating the user’s profile from multimodal embedding, we find a similar user’ *u*_*j*_ given in Eq (12) below, defining the user-user similarity. To avoid over-fitting in our KNN model, we considered relatively larger number of neighbours due to the reason that smaller number of k neighbours often lead to overfitting.


$$j\in U.sim(u,u_{j})$$ (12)

**Fig 10:**
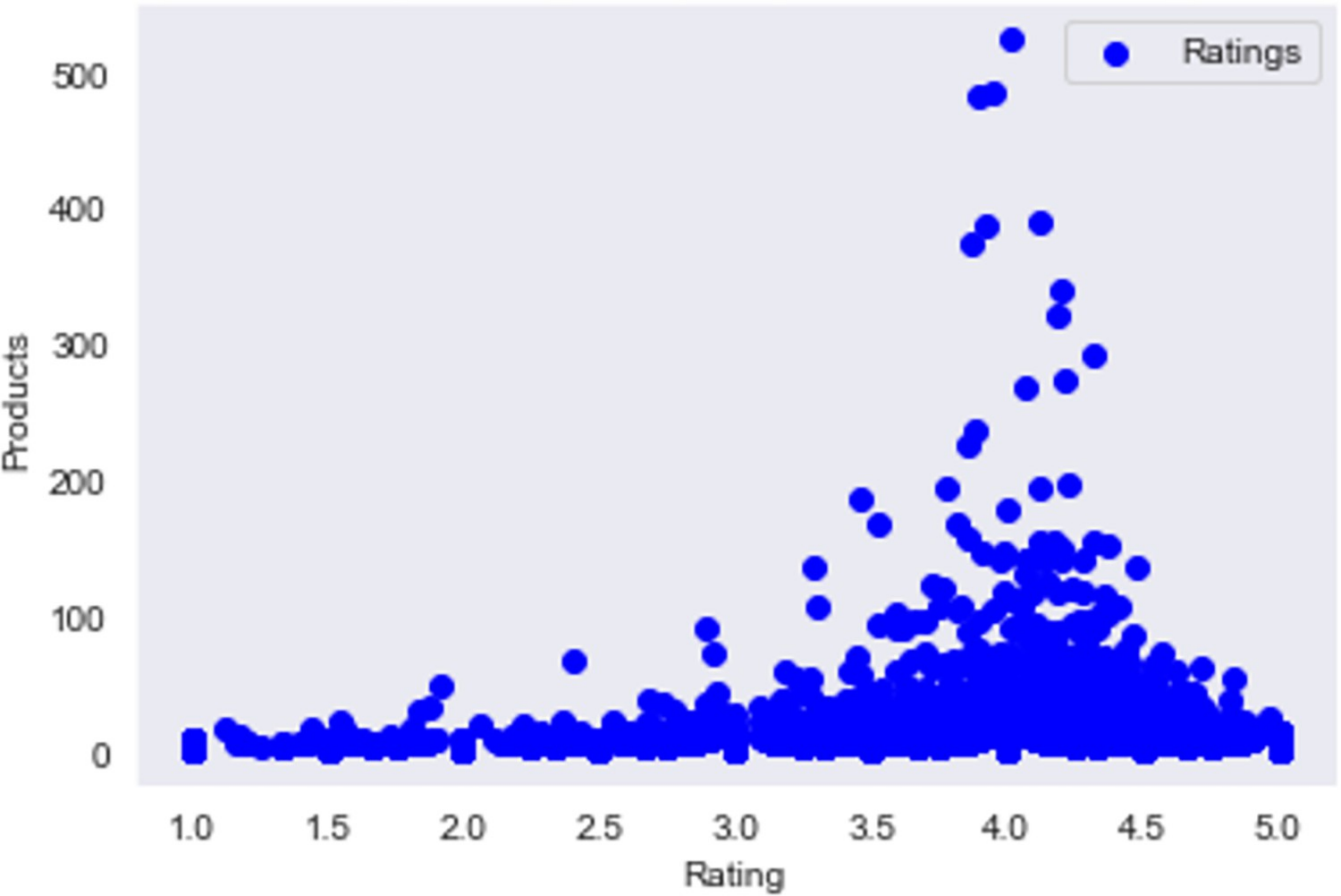
The average rating for products in BE-dataset.

We look for popular items among other similar users and recommend those items to the user *u* which he has not purchased yet. An average rating for products in BE-Dataset is shown in Fig 10 below.

As discussed earlier, a cold start is a challenge when a new user interacts with the system or a user has been in the system for a certain time but has not done anything. The SVD could only extract the vector of users and things, preventing the refinement of additional features and diminishing recommendation accuracy. Our proposed feature learning model, DTLME makes use of transfer learning and multimodal embedding networks to learn the latent features for users and items, which allows the generation of rich representations for users and items. Transfer learning enables our model to generate a similarity model for the cold-start items, which allows a more accurate prediction of relevant items to an active user. The model uses cart items and user wish-listed items information in addition to the order history and similarity matrices, to generate a specific user profile.

### 5.2 Performance analysis

In comparison with baseline recommender systems, dense similarity matrices help in generating more realistic and satisfactory recommendations. The output of the similarity criteria is relatively higher due to the use of SVD to reduce the dimensions of the concatenated vectors. Similarity clusters are produced on the basis of these matrices which allows quick mapping of an active user to a specific users’ group and thus reducing the prediction time and improving the performance of the proposed model. As a result, this hybrid feature learning model seems to be effective in alleviating the sparsity and cold start issues for users who registered recently or have been inactive so far.

**Fig 11:**
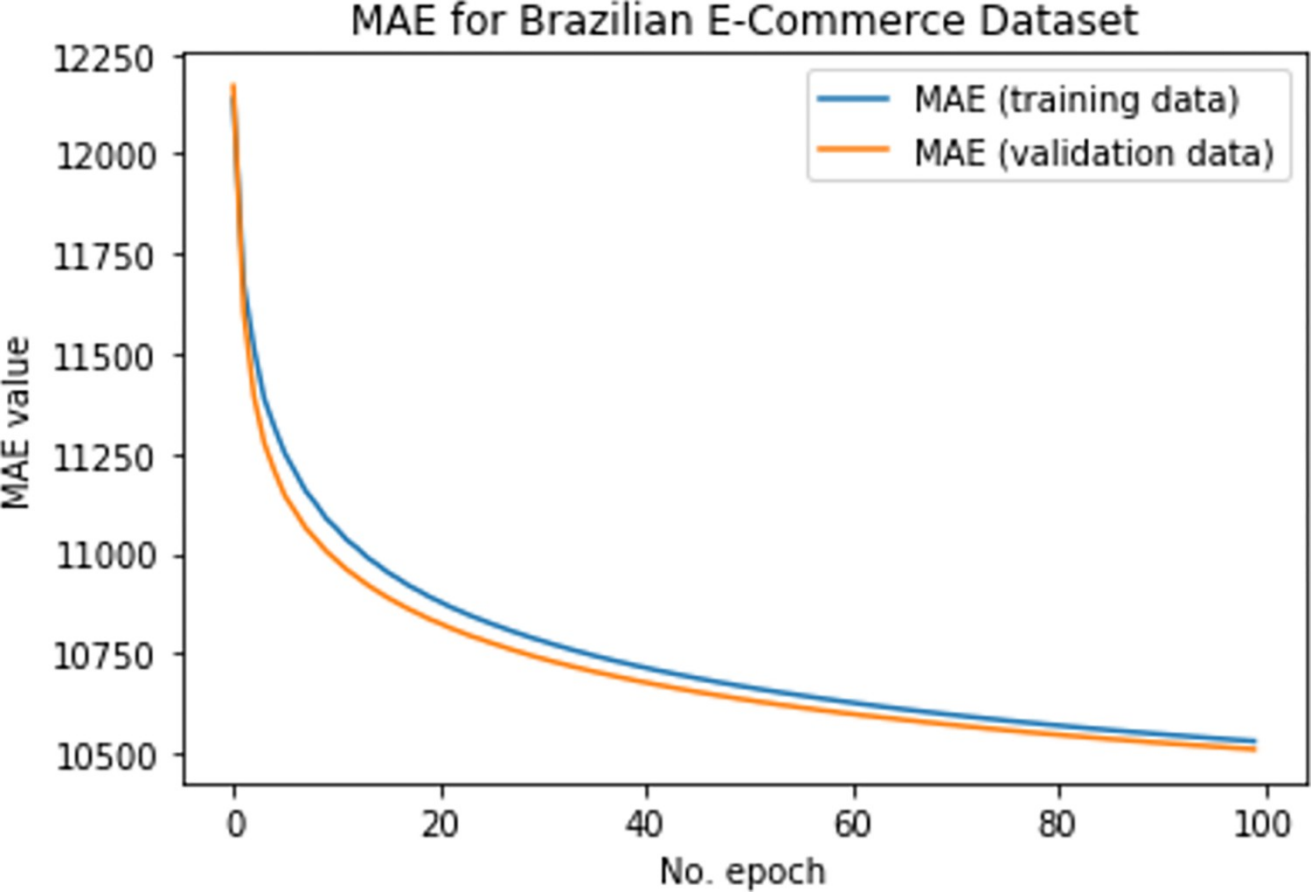
MAE for BE-dataset @100 epochs.

**Fig 12:**
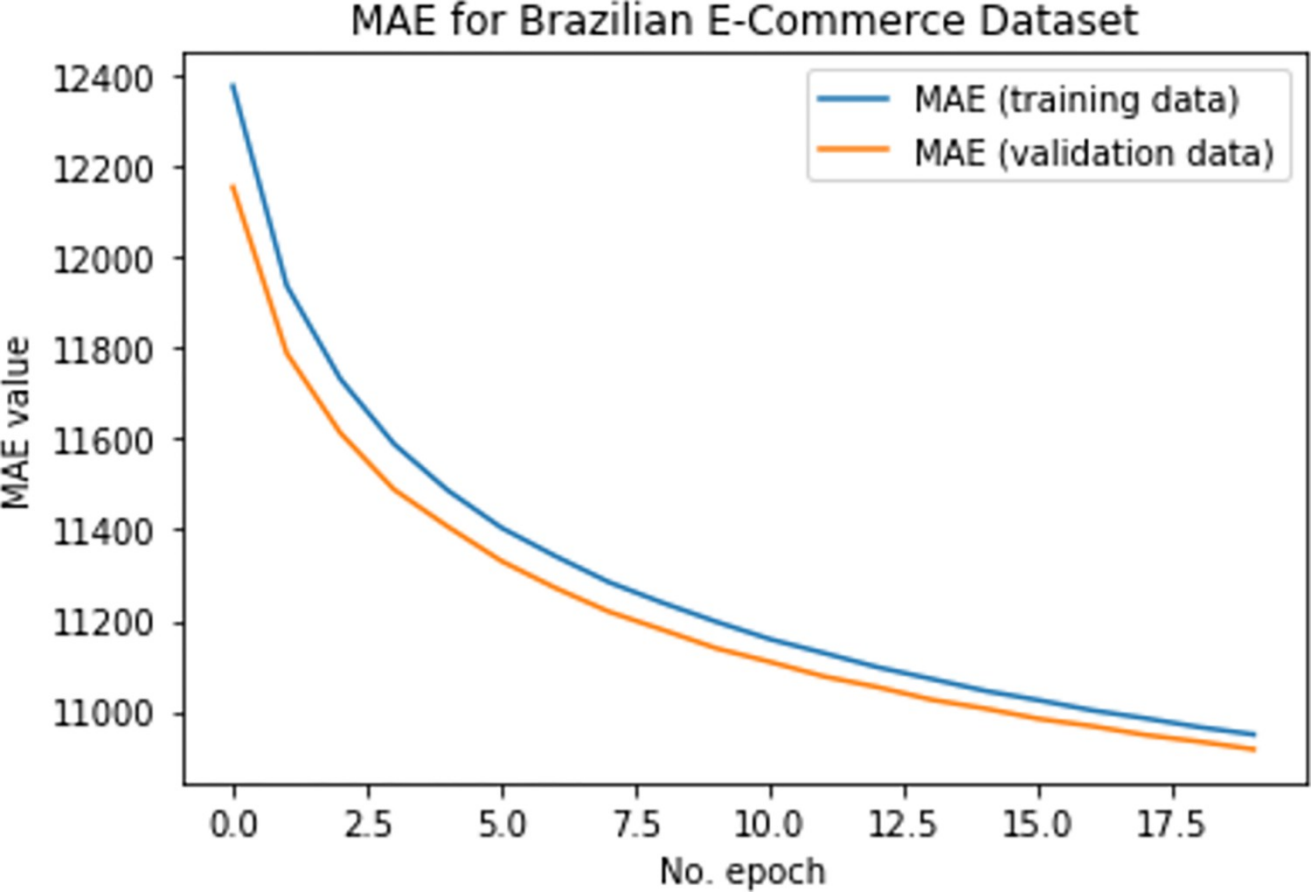
MAE for BE-dataset @20 epochs.

To assess the efficacy of the suggested DTLME method for cold start, MAE is calculated for the Brazilian e-commerce dataset, shown in Figs 11 and 12 below. Analysis shows that the DTLME produced better results with a lower error rate than other existing methods.

**Fig 13:**
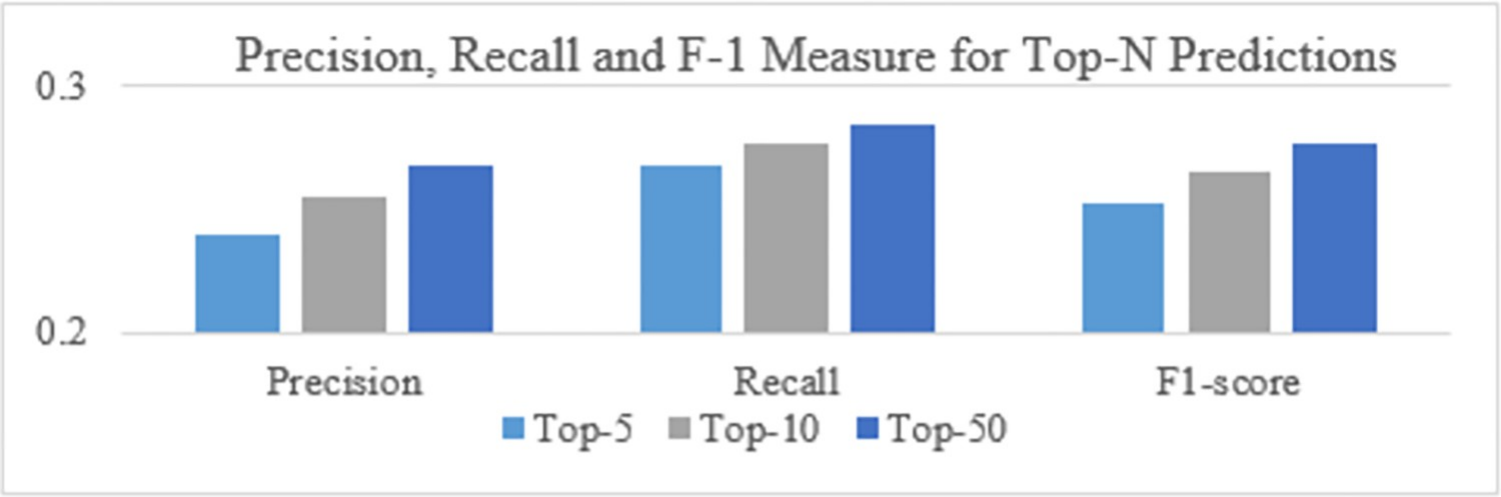
Precision, recall and F-1 measures for DTLME model.

Comparison for precision, recall and F1-score for top-N recommendations generated by the proposed DTLME model is shown in Fig 13 below. The result depicts the performance improvements for all three measurements over top-N items, predicted by the proposed model.

### 5.3 Comparison with baseline recommender systems

By adding multimodal embedding into latent components to develop better feature representations, our proposed model outperformed the baseline techniques. Direct comparison experiments were conducted to judge the performance of the suggested DTLME model when compared against CSSVD [58], TF [59], and BPR [60] algorithms. Precision, Recall, F1-score, and MAE measures were used to evaluate the performance of the specified methods. The results show that the proposed method outperformed baseline methods as shown in Table 3 below.

**Table 3:** Performance comparison of proposed model with baseline recommendation techniques.

RS	Top-N	Precision	Recall	F1-score
CSSVD	5	0.23	0.26	0.244
	10	0.247	0.27	0.258
	50	0.248	0.27	0.259
BPR	5	0.223	0.305	0.258
	10	0.22	0.294	0.252
	50	0.227	0.242	0.234
TF	5	0.218	0.29	0.249
	10	0.215	0.285	0.245
	50	0.212	0.263	0.235
DTLME	5	0.239	0.267	0.252
	10	0.255	0.276	0.265
	50	**0.268**	**0.284**	**0.276**

**Fig 14:**
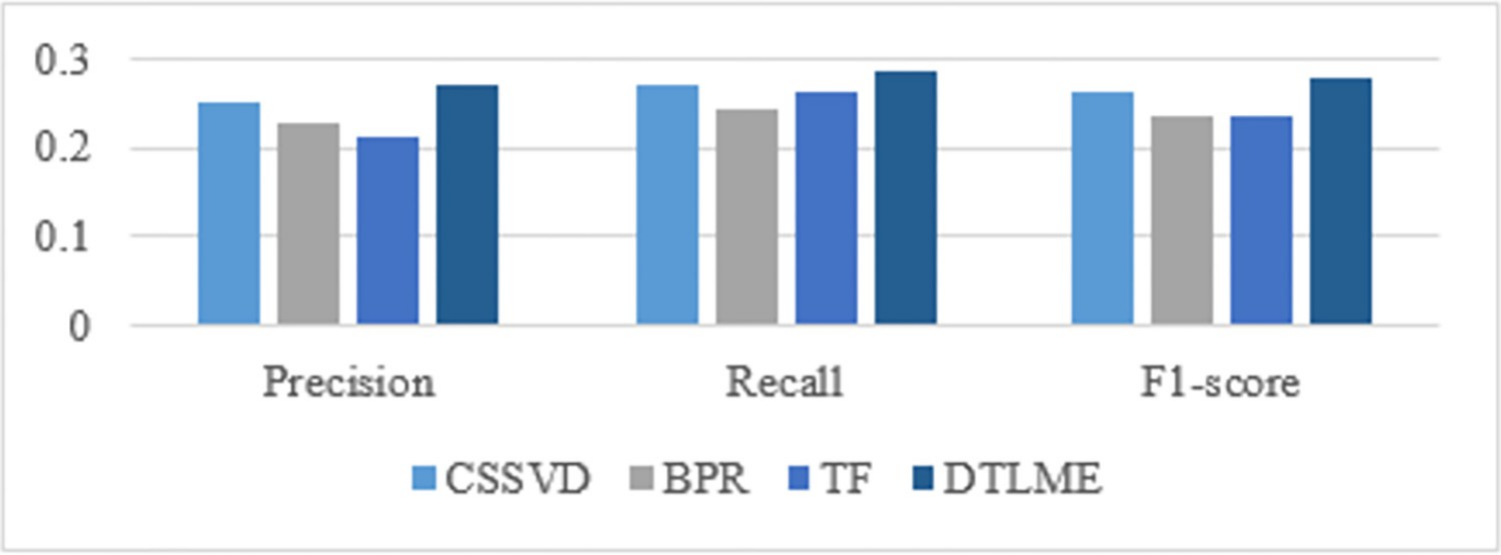
Comparative analysis of DTLME model with CSSVD, BPR and TF baseline RSs.

Proposed DTLME model achieved better results for evaluation measures of precision, recall and F-1 score in comparison to the baseline RSs, on the Brazilian e-commerce dataset (Fig 14). DTLME achieved an F1-score of 0.276, which is higher than the rest of the models in comparison. An increase in precision and F1 scores indicates that the model has improved results in recommending top-N items to the user.

### 5.4 Dealing with the sparsity

We compare our model with baseline RSs under various data sparsity rate (SR) circumstances to ensure that it is legitimate. We handle sparsity in data by modifying the training set’s size. We randomly choose ratings between 10% and 90% as our training set from the rating matrix whereas, the rest is taken as a test dataset. We select 10% of our dataset as a sample to demonstrate SR calculating method. In the user-item matrix, there are 98,410 ratings, and 10% or 9841 of the dataset is chosen as training dataset. Then sparsity can be estimated using Eq (3) as follows:

$$SR=1-\frac{8941}{2044*1290}=99.66\%$$

The sparsity rate (SR) and RMSE based comparison of the proposed model and the baseline RSs for various training sized datasets is presented in Table 4 below.

**Table 4:** Comparison of the proposed model with baseline algorithms for RMSE on the basis of SR.

Training Size	SR	RMSE			
		CSSVD	BPR	TF	DTLME
90%	96.28	0.8633	0.8438	0.8397	0.8189
80%	96.67	0.8761	0.8506	0.8456	0.8392
70%	97.04	0.8812	0.8696	0.8591	0.8412
60%	97.53	0.8921	0.8834	0.8653	0.8558
50%	97.96	0.9019	0.8923	0.8702	0.8695
40%	98.45	0.9138	0.9178	0.8958	0.8786
30%	98.79	0.9253	0.9355	0.9059	0.8943
20%	99.28	0.9342	0.9483	0.9183	0.9078
10%	99.66	0.9465	0.9565	0.9287	0.9128

Table 4 presents RMSE for the proposed and the baseline algorithms, for different SR. Results show that the suggested model outperformed baseline RSs for a variety of data sparsity situations, which could be attributable to two reasons: Firstly, a user has contributed ratings to only a few items usually, out of a larger set, resulting in a highly sparse rating matrix. And secondly, existing CF-based RSs rely solely on a user-item rating matrix, ignoring the possible attributes of both users and objects. The proposed system, on the other hand, makes use of deep transfer learning and multimodal embedding to integrate user and item-related information, in addition to the rating matrix. As a result, the proposed system can fully learn potential users and item attributes for the recommendation.

### 5.5 Dealing with the cold-start problems

CF algorithms are mostly based on user’s ratings for items and in some techniques, use user, item basic information to produce the recommendations. Only rating matrix alone doesn’t guarantee accurate predictions and therefore, results in the cold-start challenges. Information about user and items play important role in dealing with this issue.

To target the user and item cold-start problems, the proposed model uses two sub models. Firstly, DTL helps in generating rich item features and produces and dense item-item similarity matrix which as a result helps in alleviating the new item cold-start problem. An item with no rating or the one, newly added to the system is effectively classified into a certain class using the DTL model, which allows it to participate in the prediction process. We validated the effectiveness of the proposed technique for item cold-start for 10, 20, 50, and 100 new items respectively. Table 5 presents the performance analysis of the selected models in case of an item cold-start problem.

**Table 5:** Performance comparison for item cold-start problem on BE-dataset.

	MAE				RMSE			
No. of Items	10	20	50	100	10	20	50	100
TF	0.7593	0.7323	0.7212	0.7089	0.5829	0.5672	0.5574	0.5418
BPR	0.7494	0.7431	0.7229	0.7132	0.5724	0.5579	0.5532	0.5435
CSSVD	0.5425	0.5298	0.5276	0.5045	0.3548	0.3463	0.3247	0.3175
DTLME	0.5346	0.5234	0.5206	0.5139	0.3267	0.3186	0.3109	0.3022

The results indicate that the proposed model outperforms the baseline methods for both MAE and RMSE values except in one scenario consisting of 100 items, where CSSVD outperformed all other models. The result show that the DTLME model has great potential to overcome the new item cold-start problem when compared with the state-of-art RSs.

In the second part, we used multimodal embedding which allows incorporating rich user, and item features into the model from multiple input sources, and building rich user profiles to tackle new user cold-start issue. In this experiment 10, 20, 50, and 100 users were used to verify the effectiveness of our model in the user cold-start condition. A comparison between the proposed model and the baseline RSs is shown in Table 6.

**Table 6:** Performance comparison for user cold-start problem on BE-dataset.

	MAE				RMSE			
No. of Items	10	20	50	100	10	20	50	100
TF	0.7683	0.7508	0.7451	0.7333	0.5892	0.5842	0.5689	0.5721
BPR	0.7454	0.7353	0.7229	0.7161	0.5752	0.5634	0.5498	0.5408
CSSVD	0.6825	0.6797	0.6743	0.6517	0.4415	0.4344	0.4274	0.4216
DTLME	0.6271	0.6122	0.6024	0.5882	0.4327	0.4275	0.4169	0.4011

It can be observed from the results presented in Table 6, that the proposed DTLME model has effectively utilized the multimodal embedding to improve the performance and accuracy of the predictions for the new users in the system. Proposed model scored 0.5882 for MAE against 100 users which is much lower than that of CSSVD which scored 0.6517. Similarly RMSE score of our model was again lower than the CSSVD model which shown that the model was able to achieve improved accuracy over the baseline RSs.

## 6 Conclusion and future work

Recommender systems are methods for dealing with large-sized data by filtering it and then presenting the user with a piece of helpful information. In this research, a new hybrid strategy is proposed to tackle the sparseness and cold-start difficulties during the online recommendation process. The proposed method DTLME combines deep transfer learning and multimodal embedding to produce dense similarity matrices for users and items. Introducing the side information like social network embedding, user sessions information, purchase history, wish-listed items, cart information and user preferences along with the user, item embedding and rating matrix allowed the model to produce more accurate user profiles. Previous research in this sector reveals that existing RSs take limited auxiliary information, whereas we combine deep transfer learning and multimodal embedding to improve the recommendation process. Integrating visual features and multimodal embedding allows the items to participate in the final list of recommendations in addition to user-based similarity predictions, thus, targeting both, item cold-start and user-cold start problems concurrently. Experimental analysis and results show that our proposed model outperforms other similarity-based RSs in terms of accuracy and performance improvements for both, sparsity and cold-start challenges.

However, despite the model’s improved performance, there are a few limitations which we aim to target in our future work. Firstly, though the proposed algorithm has performed better than the baseline methods, however, it still has issues with time and memory utilization due to a relatively larger volume of side information being considered as compared to existing models. Secondly, we have targeted BE-Dataset in this study, but it can be expanded to experiment the proposed model with other widely used datasets, like MovieLens, RetailRocket and Yelp datasets. Moreover, neural network techniques may also be integrated during the user profile generation process to experiment the model’s performance.
